# Resistance to anticoagulant rodenticides in Martinique could lead to inefficient rodent control in a context of endemic leptospirosis

**DOI:** 10.1038/s41598-019-49661-5

**Published:** 2019-09-17

**Authors:** Aurélie Marquez, Rami Abi Khalil, Isabelle Fourel, Teddy Ovarbury, Adrien Pinot, Armand Rosine, Gérard Thalmensi, Georges Jaffory, Angeli Kodjo, Etienne Benoit, Virginie Lattard

**Affiliations:** 1 USC 1233 RS2GP, VetAgro Sup, INRA, Univ Lyon, F-69280 Marcy l’étoile, France; 2 FREDON Martinique, Route du Lycée agricole, Chemin Tolobe, Croix Rivail, 97224 Ducos, Martinique; 3 ARS Martinique, Centre d’affaires AGORA, Zac de l’Etang Z’abricot, Pointe des grives, CS 80 656, 97263 Fort de France Cedex, Martinique; 4 UMR 0874 UREP, VetAgro Sup, INRA, Univ Clermont, Clermont-Ferrand, France

**Keywords:** Ecology, Epidemiology

## Abstract

Leptospirosis is a re-emergent worldwide zoonosis. It is endemic in Martinique where transmission conditions are favourable. Humans are usually infected through contact with water contaminated with urine of rodents. Recent human leptospirosis outbreaks in Martinique require today effective rodent management to prevent leptospirosis transmission. Nowadays, use of anticoagulant rodenticides (AR) is the main method implemented to control rodent populations. Nevertheless, intensive use of these AR has selected worldwide many VKORC1-based resistant rodent strains to AR. Our aim was to characterize the sensitivity of Martinique commensal rodents to AR to better prevent leptospirosis transmission. Resistance of house mice to first-generation and in rare cases even to second-generation ARs were clearly demonstrated in Martinique with the detection of the Y139C mutation with a very high allelic frequency of 40% and the A26T/Y139C double-mutation with an allelic frequency of 0.9%. In black rat, the most prevalent rodent in Martinique, 3 new *Vkorc1* coding mutations were detected, the H68N, A115T and S149N mutations associated with moderate resistance to first generation AR. Therefore, rodent management in Martinique must be carried carefully to avoid resistance diffusion and maintain long-term effective rodent management, to be able to efficiently prevent leptospirosis transmission.

## Introduction

Leptospirosis, a bacterial disease, is one among the foremost widespread zoonoses within the world^1,2^. This disease has been reported to affect on more or less one million persons worldwide every year^3^ and may be accompanied by different clinical symptoms ranging from a flu-like syndrome with a moderate fever to a multi-organ failure^4–6^ which can lead to death^7^. Incidence of this disease is much higher in the tropics than in the temperate zones, the transmission conditions being more favorable^1^. In Martinique, an Island of the French West Indies, incidence was reported at 13.9 to 60.6 cases per 100 000 inhabitants per year^8,9^ with frequent outbreaks^10,11^. All preventive measures must be implemented to limit leptospirosis transmission in Martinique. Even if numerous wild and domestic animals are known as as hosts of pathogen *Leptospira* excreting them in their urine, the main reservoirs identified are rodents, hence the importance of managing their populations^12,13^.

Nowadays, use of anticoagulant rodenticides ARs is the main method implemented to control rodent populations. They provoke bleeding by the inhibition of the vitamin K epoxide reductase enzyme (VKORC1). This enzyme catalyses the reduction of vitamin K epoxide into vitamin K quinone permitting the vitamin K recycling. Vitamin K is useful as a cofactor of the gamma -glutamyl carboxylase enzyme that activates coagulation factors II, VII, IX and X by gamma-carboxylation. Anticoagulant rodenticides (AR), by inhibiting VKORC1, prevents the coagulation process^14,15^.

The massive use of ARs since the 1960s selected rodents resistant to the first-generation ARs (i.e., warfarin, chlorophacinone, diphacinone, coumatetralyl)^16,17^. Metabolic resistance caused by a faster elimination of ARs has been reported. Nevertheless, resistance to ARs is mainly due to Vkorc1 mutations or polymorphisms^18–21^ resulting in a decrease of the inhibition of VKORC1 enzyme by first-generation ARs. Such resistance has been first described in brown rats (*Rattus norvegicus)* and then in house mice (*Mus musculus domesticus)* in Europe, and then worldwide in the United States^22^, Canada^23^, Japan^24^ and in Australia^25^…. The selection of rodents with *Vkorc1* mutations has led to an inefficiency of first-generation AR to control certain rodent populations. Second generation ARs -i.e., bromadiolone, difenacoum, difethialone, brodifacoum and flocoumafen) have been thus developed to overcome resistance issues in rodents. Because second generation ARs are persistent in rodent tissues and related to secondary exposure or poisoning of non-target wildlife, their use should be cautious and sometimes, in some countries, limited to professionals and/or after failure of a first-generation AR treatment and/or if resistance is demonstrated.

In Martinique where leptospirosis is so prevalent, rodent management must be optimal. This study was therefore intended to evaluate the presence and dispersion of *Vkorc1* mutations in rodents in Martinique that could impair rodent management by ARs. To evaluate the impact of the detected *Vkorc1* mutations and because all ARs can be used in Martinique as biocides and sometimes as plant protection products, exposition of rodents to ARs was characterized.

## Results

### Trapping success

A total of 512 traps were set in Martinique. The trap effort on the Martinique Island all along the study was 2048 trap-nights. The trap effort was different according to the site: 88 trap-nights for site 8, 100 for sites 1 to 6, 108 for site 7, 192 for site 9, 200 for sites 10 to 13, and 260 for site 14. A total of 206 rodents were captured (129 *Rattus rattus*, 59 *Mus musculus domesticus* and 18 *Rattus norvegicus*). Rodent species were confirmed based on matching of mtDNA cytochrome b sequences with more than 98% of homology with published sequences of the *cytochrome b* of the respective species. Trapping results per site are presented in Table 1. The trap success was 10.06 rodents per 100 trap-nights in Martinique Island including 6.30 *Rattus rattus*, 2.88 *Mus musculus domesticus* and 0.88 *Rattus norvegicus*. Results per site are presented in Table 2 with trap-success ranging from 0 rodent for site 4 to 23.5 rodents for the control site 14. The trap-success for *Rattus rattus* was comprised between 0 to 23.5 for site 14; for *Mus musculus*, between 0 to 12 (for site 5); for *Rattus norvegicus*, between 0 to 10 (for site 2).

**Table 1:** Trapping details of rodents in Martinique Island. Between brackets, is indicated the number of samples analyzed.

Dates of trapping	Site	Activity	Number of traps	Trapped rodents		
				Rr	Mm	Rn
From 04/13/15 to 04/17/15	Site 1	Agricultural (sugar cane)	25	5	0	0
	Site 2	Agricultural (sweet potato, cassava)	25	1	0	10 (8)
From 06/15/15 to 06/19/15	Site 3	Intensive farming (poultry)	25	1	0	1
	Site 4	Intensive farming (poultry and pig)	25	0	0	0
From 07/20/15 to 07/24/15	Site 5	Agricultural (cassava)	25	4	12	0
	Site 6	Agricultural (cassava)	25	1	9 (8)	0
From 08/31/15 to 09/04/15	Site 7	Agricultural (banana tree)	27	5	4	0
	Site 8	Agricultural (banana tree)	22	12	1	1
From 09/14/15 to 09/18/15	Site 9	Control forest area (as control site)	48	23	0	1
From 09/21/15 to 09/25/15	Site 10	Agricultural (chayote)	50	5 (4)	2 (1)	2
From 10/12/15 to 10/16/15	Site 11	Industrial and commercial area	50	5	8 (6)	1
From 10/19/15 to 10/23/15	Site 12	Industrial and commercial area	50	0	14	0
From 11/16/15 to 11/20/15	Site 13	Agricultural (sugar cane)	50	6	9	2
From 11/23/15 to 11/27/15	Site 14	Control natural area (as control site)	65	61 (14)	0	0
**Total**			**512**	**129**	**59**	**18**

**Table 2:** Trap-success per site and per species.

	Total rodents Rr + Mm + Rn	*Rattus rattus* (Rr)	*Mus musculus* (Mm)	*Rattus norvegicus* (Rn)
Site 1 (ARs site)	**5.00**	5.00	0.00	0.00
Site 2 (ARs site)	**11.00**	1.00	0.00	10.00
Site 3 (ARs site)	**2.00**	1.00	0.00	1.00
Site 4 (ARs site)	**0.00**	0.00	0.00	0.00
Site 5 (ARs site)	**16.00**	4.00	12.00	0.00
Site 6 (ARs site)	**10.00**	1.00	9.00	0.00
Site 7 (ARs site)	**8.33**	4.63	3.70	0.00
Site 8 (ARs site)	**15.91**	13.64	1.14	1.14
Site 9 (control site)	**12.50**	11.98	0.00	0.52
Site 10 (ARs site)	**4.50**	2.50	1.00	1.00
Site 11 (ARs site)	**7.00**	2.50	4.00	0.50
Site 12 (ARs site)	**7.00**	0.00	7.00	0.00
Site 13 (ARs site)	**8.50**	3.00	4.50	1.00
Site 14 (control site)	**23.46**	23.46	0.00	0.00

### Exposition of trapped rodent populations to ARs

**Figure 1:**
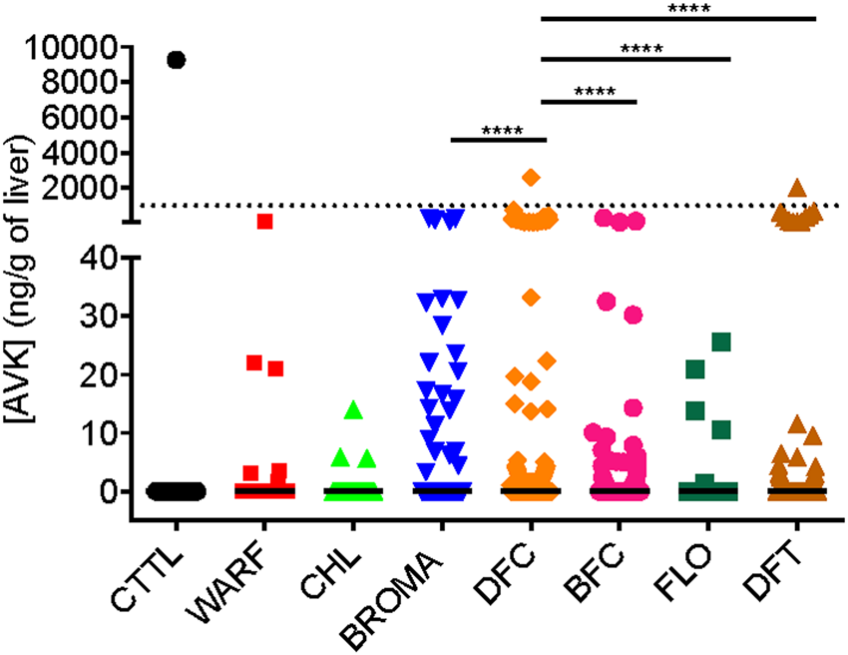
AR concentration in liver of trapped rodents in Martinique Island. Concentrations of individual (**A**) or grouped in first or second generation (**B**) AR molecules are presented. AR concentrations were determined by LC-MS/MS as previously described^47^. Results are presented as median with interquartile range. First-generation AR = CTTL, coumatetralyl; WARF, warfarin; CHL, chlorophacinone. Second-generation AR = BROMA, bromadiolone; DFC, difenacoum; BFC, brodifacoum; FLO, flocoumafen; DFT, difethialone. The dashed line corresponds to the concentration of 1000 ng/g.

**Figure 2:**
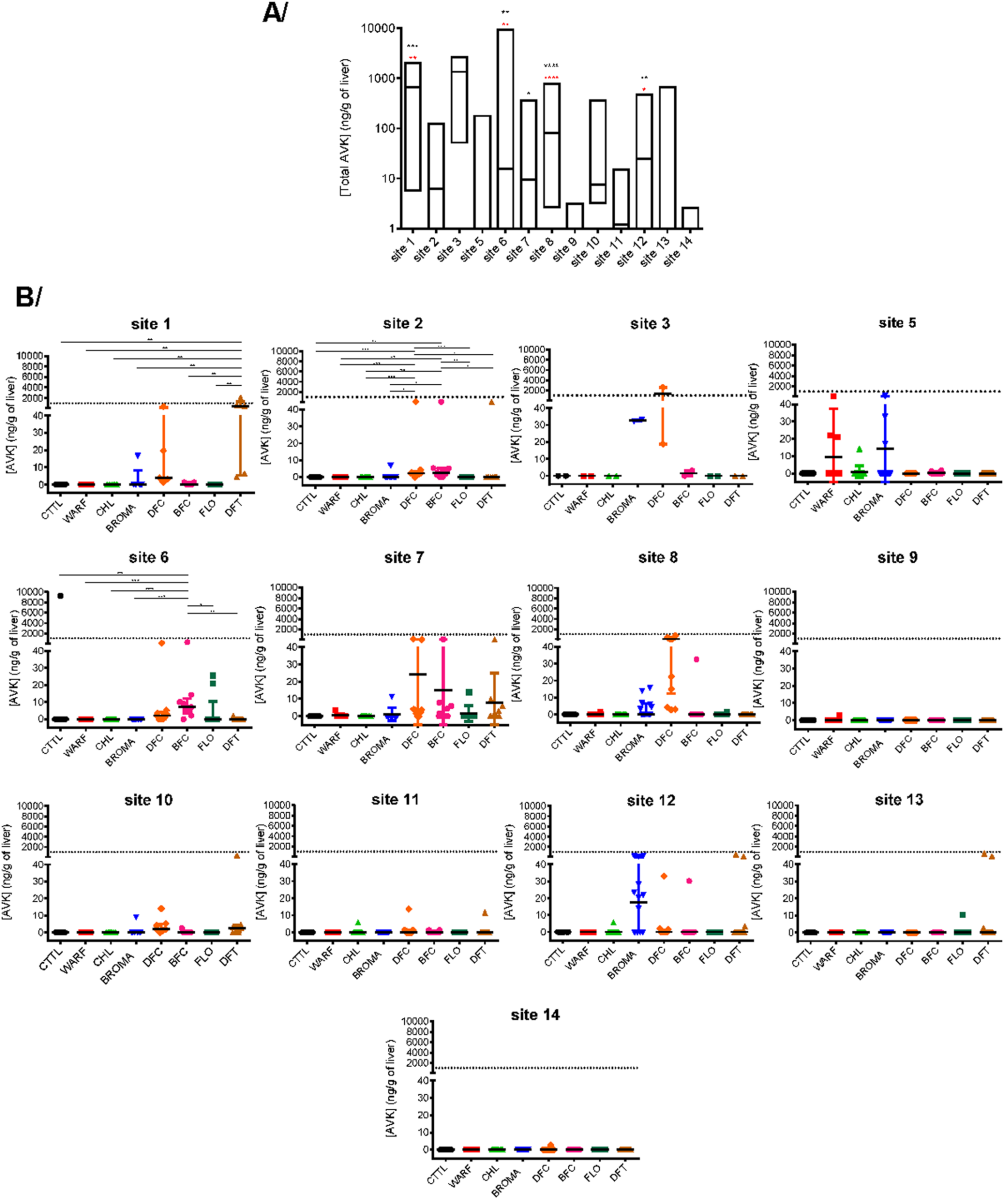
AR concentrations in liver of rodents trapped in the different trapping sites. Concentrations of total (**A**) or individual (**B**) ARs are presented. AR concentrations were determined by LC-MS/MS as previously described^47^. Results are presented in (**A**) as median with a box extending from the 25th to 75th percentiles, in (**B**) as median with interquartile range. Sites 9 and 14 corresponded to control sites. First-generation AR = CTTL, coumatetralyl; WARF, warfarin; CHL, chlorophacinone. Second-generation AR = BROMA, bromadiolone; DFC, difenacoum; BFC, brodifacoum; FLO, flocoumafen; DFT, difethialone. The dashed line corresponds to the concentration of 1000 ng/g. RR for Rattus rattus, MM for Mus musculus and RN for Rattus norvegicus.

Rodent exposure to ARs was assessed by analyzing residues of ARs in liver. The rodents trapped in control sites 9 and 14 did not have hepatic residues of ARs. In the other sites, 61 rodents presented liver ARs residues of less than 10 ng/g (54% of rodents trapped in ARs sites); 26 rodents, concentrations between 10 and 100 ng/g (or 23% of rodents trapped in ARs sites); 23, concentrations between 100 and 1000 ng/g (or 20% of rodents trapped in ARs sites); and 3, concentrations greater than 1000 ng/g (*i.e*., 2.6% of rodents trapped in ARs sites). The majority of ARs residues were molecules belonging to second-generation ARs with difenacoum residues significantly higher than other second-generation molecules (Figs 1, 2B).

**Figure 3:**
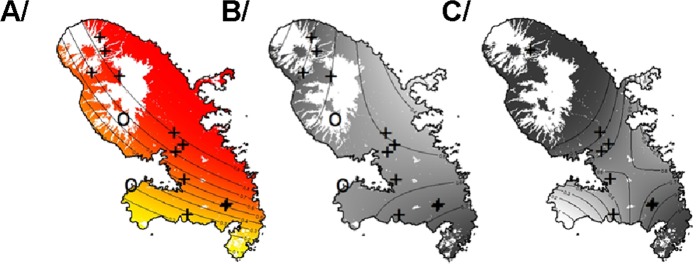
Estimation of the proportion of individuals exposed to ARs (**A**), the proportion of mutants in rat population (**B**), the proportion of mutants in mouse population (**C**). A Generalized Linear Model as a smoothing model was used to analyze the geographical pattern. Binomial distribution for the response variable (proportion of rodent with ARs or proportion of mutated rodents) and logit link were used. The coordinates X and Y was used as explanatory variables. Proportion of rodent exposed to ARs increases from yellow to red. Proportion of mutant rodents increases from grey to black. White areas represent natural area where AVK treatments are unexpected. “0” symbols represent locations of sampled sites in natural area (control sites), “ + ” symbols, those in other locations. Note that map of mouse model (**C**) is adjusted on a smallest number of sites than (**A**) and (**B**) and that estimated coefficients (Xlong, Ylat and interaction between Xlong and Ylat) are less accurate.

A Generalized Linear Model as a smoothing model was used to analyze the geographical pattern of ARs exposition. The best model selected by its AIC (Akaike information criterion) used the latitude, the longitude, an interaction between latitude and longitude and the nature of the site (natural area or not) as variables. All these parameters were highly statistically significant. Spatial variables (Ylat, Xlong and interaction between Ylat and Xlong) revealed a strong spatial pattern with low exposure in South-west to a high exposure to Northeast (Fig. 3A). Furthermore, as expected, sites in natural area showed significant lower AVK exposure.

Whether it was agricultural site or industrial site, residues of ARs were similar (result not shown), even if there were noticeable differences between agricultural sites, with highly treated sites such as sites 1 and 8 (significantly different from the control sites) and weakly treated sites such as site 13 (not significantly different from the control sites) (Fig. 2A).

**Figure 4:**
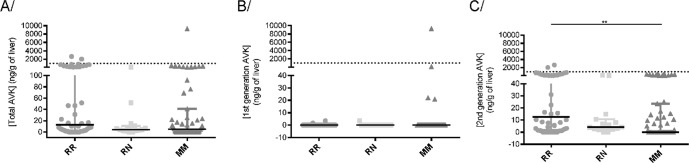
AR concentrations in liver of trapped Rattus rattus (RR), Rattus norvegicus (RN) and Mus musculus (MM). Concentrations of total (**A**), first generation (**B**), and second generation (**C**) ARs are presented. AR concentrations were determined by LC-MS/MS as previously described^47^ First-generation AR = coumatetralyl + warfarin + chlorophacinone. Second-generation AR = bromadiolone + difenacoum + brodifacoum + flocoumafen + difethialone. The dashed line corresponds to the concentration of 1000 ng/g.

ARs residues were detected in *Rattus rattus*, *Rattus norvegicus* and *Mus musculus* (Fig. 4). First generation ARs were detected in concentration higher than 5 ng/g only in liver of *Mus musculus*. Second generation AR were detected in all species but were significantly higher in liver of *Rattus rattus* than *Mus musculus*. No difference in concentration was observed between mutated and non-mutated rodents (data not shown) even when only one mutation was considered to perform this correlation. On the other hand, a positive correlation was observed between the occurrence of mutants in rats [0.17372, +/− 0.01346] and mice [0.11303 +/− 0.03465] population and the occurrence of ARs.

### Vkorc1 genotyping of trapped rodent populations

From the sampling, *Vkorc1* of 81 *Rattus rattus*, 55 *Mus musculus* and 16 *Rattus norvegicus* have been sequenced. From *Mus musculus*, all the sequences were exploitable. From *Rattus sp*., only *Vkorc1* sequences of 61 *Rattus rattus* and 15 *Rattus norvegicus* were exploitable. 34 *Rattus rattus* presented no silent or missense Vkorc1 mutation in the coding sequence (*i.e*., 55.7% of the sequenced samples from *Rattus rattus* species) and 4 presented missense mutations in the coding sequence (*i.e*., 6.6% of the sequenced samples from *Rattus rattus* species). One *Rattus norvegicus* presented no silent or missense Vkorc1 mutation in the coding sequence, the others presented silent mutations. Only 21 Mus musculus presented no silent or missense *Vkorc1* mutation in the coding sequence (*i.e*., 38.2% of the sequenced samples from *Mus musculus* species) and 34 presented missense mutations (*i.e*., 61.8% of the sequenced samples from *Mus musculus* species).

**Figure 5:**
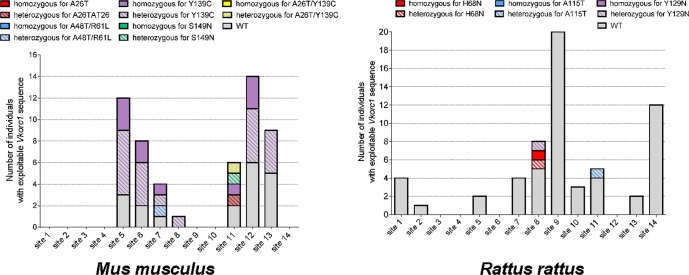
Distribution of Vkorc1 SNPs or mutations per site in trapped *Rattus rattus* and *Mus musculus*. Presence of *Vkorc1* SNPs or mutations was analyzed by PCR-amplification and sequencing of the *Vkorc1* gene.

Among sequenced *Rattus rattus*, 6 different SNPs or mutations found either alone or in combination were detected including 3 silent mutations L94L (g.1087 C > T), S120S (g.2005A > T), A143A (g.2083 A > G) and 3 missense mutations H68N (g.1009 C > A), A115T (g. 1997G > A), Y129N (g.2039 T > A). Allelic frequencies of the different genotypes are presented in Table 3 and locations of genotypes leading to protein mutations are presented in Fig. 5.

**Table 3:** *Vkorc1* genotypes found in rodents in Martinique Island.

Species	Nucleotide mutation	Protein mutation	Exon	Allelic frequency in rodents sampling
*Mus musculus*	G76A	A26T	1	0.9
	G142A/G976T	A48T/R61L	1/2	1.9
	A2223G	Y139C	3	39.6
	G76A/A2223G	A26T/Y139C	1/3	0.9
	G2253A	S149N	3	0.9
*Rattus rattus*	C1009A	H68N	2	2.6
	C1087T	L94L	2	18.1
	G1997A	A115T	3	0.9
	A2005T	S120S	3	1.7
	T2039A	Y129N	3	0.9
	C1087T/A2083G	L94L/A143A	2/3	6.0
*Rattus norvegicus*	C1140T	H68H	2	93.3

Among sequenced *Rattus norvegicus*, only 1 silent mutation H68H (g.1140 C > T) was detected with an allelic frequency of 93.3%. This silent mutation was found in sites 2, 3, 8, 9, 10, 11 and 13. A Generalized Linear Model as a smoothing model was used to analyze the geographical pattern of the proportion of mutations in rats. The best model selected by its AIC used the latitude, the longitude, an interaction between latitude and longitude and the nature of the site (natural area or not) as variables. All these parameters were highly statistically significant. Spatial variables (Ylat, Xlong and interaction between Ylat and Xlong) revealed a strong spatial pattern with a proportion of rats with *Vkorc1* mutations increasing highly increased to North and South and less in West (Fig. 3B). Furthermore, as expected, sites in natural area showed significant lower proportion of mutants.

Among sequenced *Mus musculus*, 5 different SNPs or mutations found either alone or in combination were detected. All these mutations were missense mutations A26T (g.76 G > A), A48T (g.142 G > A), R61L(g.976 G > T), Y139C(g.2223 A > G) and S149N(g.2253 G > A). Allelic frequencies of the different genotypes are presented in Table 3 and locations of genotypes leading to protein mutations are presented in Fig. 5. A spatial analysis was also carried out but due to the small number of samples and sites, the criteria used were not statistically significant.

### Functional consequences of VKORC1 mutations

**Figure 6:**
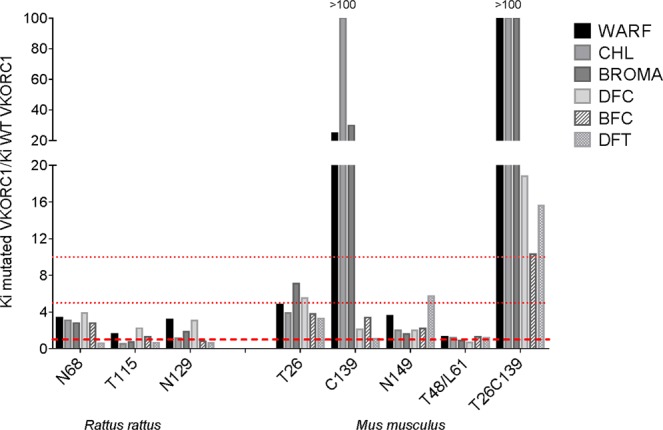
Resistance factors towards various ARs molecules of mutated VKORC1 detected in trapped rodents on Martinique Island. Resistance factor correspond to the ratio between Ki for mutated VKORC1 and wild type VKORC. Ki was the inhibition constant determined by expressing and characterising the susceptibility to AR of the recombinant mutated or non mutated VKORC1 enzyme. First-generation AR = WARF, warfarin; CHL, chlorophacinone. Second-generation AR = BROMA, bromadiolone; DFC, difenacoum; BFC, brodifacoum; DFT, difethialone. The dashed lines correspond to the resistance factor of 1, 5 and 10.

To evaluate the consequences of VKORC1 mutations on the susceptibility of VKORC1 enzyme towards AR, the unknown VKORC1 mutants were overexpressed as c-myc-fused proteins in *P. pastoris*; wild type VKORC1 of *Rattus sp*. or *Mus musculus* and some mutants (i.e., *Mm*VKORC1-A26T and *Mm*VKORC1-Y139C) having been previously characterized (Goulois *et al*., 2016, 2017, Hodroge *et al*., 2011). *P. pastoris* were able to efficiently expressed all the VKORC1 mutants with the same molecular weight of approximately 20-kDa. Respective inhibition constants (*K*_i_) towards AR of first generation (i.e., warfarin, chlorophacinone) or second generation (i.e., bromadiolone, difenacoum, difethialone or brodifacoum) were determined. Resistance factors of the mutated VKORC1 are presented in Fig. 6. This resistance factor corresponds to the ratio between obtained *Ki* for the mutated VKORC1 and obtained *Ki* for the wild type VKORC1. Previous results obtained for *Mm*VKORC1-A26T and *Mm*VKORC1-Y139C in previous studies have been added to allow the comparison.

## Discussion

This study is the first study reporting rodent resistance to ARs in Martinique. This finding is crucial to improve their management in this island where leptospirosis is an endemic disease^9^. Rodents are central in the transmission of leptospirosis. This portage has been widely described in brown rats (*Rattus norvegicus*) in the French West Indies, but it has been also reported in black rats (*Rattus rattus*) and domestic mice (*Mus musculus*)^26,27^. The management of these 3 species of rodents is therefore vital to limit leptospirosis in the French West Indies. This study aimed therefore to explore practices and effectiveness of rodent chemical treatments in Martinique.

The study aimed to be as representative as possible of the island with an important number of study sites (*i.e*., 14 sites) distributed homogeneously on the island (*i.e*., 5 sites in the North, 4 sites in the Center and 5 sites in the South), representing all the sectors of activity (*i.e*., 2 industrial / commercial sites, 2 intensive breeding sites, 2 forest/natural areas, 8 agricultural areas of sugar cane, cassava, banana, sweet potatoes or chayotes), since rodents present and practices may be different depending on the sector. 206 rodents were captured in this study, this must be analyzed considering to the trapping effort. For this, the trap-success index, which reflects the population levels, has been calculated. This success trap, including all the sites and the 3 species of rodents is 10.06 rodents/100 trap nights. A recent study conducted in a Parisian park in which rats were known to be abundant as many visitors were reporting rat sightings at day time, pointed out a trap success between 0.32 and 1.58^28^. The population of rodents in Martinique would therefore be quite abundant, especially since all the sites except the control ones are sites on which rodent management is organized. In our study, brown rats, black rats, and house mice were trapped. These 3 species are therefore present in Martinique, nevertheless the black rat seems most abundant (*i.e*., trap success of 6.3), then the domestic mouse (*i.e*., trap success of 2.9), the brown rat population (*i.e*., trap success of 0.9), being rather limited. Nevertheless, this distribution varies according to the site.

The management of brown rats, black rats and house mice in Martinique, as in the rest of the French West Indies, is largely based on the use of rodenticide anticoagulants. In our study, 54% of rodents were exposed to at least one AR molecule, 29% of which were exposed to more than 2 molecules, confirming this preferential use of ARs in rodent management and suggesting the use of several types of active ingredients on the same site. First- and second-generation AR were detected in rodents trapped in urban as well as agricultural sites. Second-generation molecules were most frequently detected (in the liver of 50% of rodents) while first-generation molecules were very rarely detected (in the liver of only 6% of rodents). Difenacoum was the most frequently detected molecule in 38% of rodents; nevertheless, hepatic concentrations were higher than 100 ng/g, threshold compatible with the death of the animal in only 7% of the cases. Bromadiolone, brodifacoum and difethialone were detected with similar respective frequencies of 15.5, 20 and 14.5%. This intense use of second-generation molecules in agricultural areas is surprising because French regulations provide for restricted use of ARs. According to the French regulation, ARs can be used as biocides - in and around the buildings for the control of the populations of commensal rodents - or as plant protection products - in field for the control of voles. Currently, 8 active ingredients are authorized as biocides (i.e., warfarin, coumatetralyl, chlorophacinone, bromadiolone, difenacoum, brodifacoum, difethialone, flocoumafen), whereas only bromadiolone is authorized for the control of voles. Nevertheless, because of the high leptospirosis risk, a collective management of brown rats, black rats and mice, subjected to a preliminary prefectural authorization, is organized every year by the FREDON in Martinique and foresees a wider use of the ARs in the cultures and Border Fields (www.martinique.pref.gouv.fr). This may explain this important detection of ARs in agricultural areas. Until now, ecotoxicity issues associated to the use of ARs have not been reported in Martinique.

Among the 14 trapping sites, some sites seem to use few or no rodenticide treatment (*i.e*.,, sites 2, 5, 6, 7, 9, 10, 11, 13, 14) with a median for total ARs contents less than 10 ng/g in liver of trapped rodents. Two of these are the control sites (i.e., sites 9 and 14) confirming the non-use of ARs in forest/natural areas; six are agricultural sites (*i.e*.,, sites 2, 5, 6, 7, 10 and 13) and correspond to sites in which rodent populations appear, not surprisingly, to be rather abundant with a trap success greater than 8 rodents per 100 trap-nights, except for site 10; the last is an industrial/commercial site. Sites 1 and 3 seem to use frequently ARs treatment with a median hepatic concentration for total ARs greater than 600 ng/g. However, the rodent population on site 1 still seems abundant with a trap-success of 5 rodents for 100 trap-nights.

The intense use of ARs in Martinique is a factor that may favor the selection of resistant strains due to mutations of the *Vkorc1* gene. This resistance has been widely reported in Europe^20,29–32^ but also worldwide, in brown rats and house mice and also more recently in black rats^24,33–35^. This study demonstrates that resistance of rodents to ARs is present in Martinique.

Despite completely independent analyses, the same global geographical pattern for mutant distribution in rat and mouse population was observed (Fig. 3B,C) with an increasing occurrence toward the north and the south of the island. A positive correlation was observed between occurrence of AR residues and occurrence of mutant in rats and mice populations suggesting an influence of ARs use. Nevertheless, the geographical pattern for ARs exposition was not similar with a low exposure in South-west to a high exposure to Northeast. (Fig. 3A) This analysis should be performed considering each AR molecule individually. Unfortunately, the limited number of samples did not allow this analysis.

This resistance is evident for the house mouse with the detection of the Y139C mutation in Martinique. This mutation has been reported to induce a very strong resistance to first generation molecules and some second generation molecules such as bromadiolone^29^ and to some extent to difenacoum based on results obtained in rats^36^. The allelic frequency of this mutation is 40% in our sampling with 10 homozygous and 21 heterozygous mice. This allelic frequency is much greater than that reported during the resistance study conducted recently in France^29^. As this mutation is present on all mice trapping sites, the use of appropriate second-generation molecules (i.e., difethialone, brodifacoum or flocoumafen) is required to control mouse populations in Martinique. Nevertheless, these molecules must be used in a carefully way and by professionals informed of the risks related to their use. Moreover, their use could be conditioned by a preliminary genotyping to minimize any ecotoxicity problem.

Other mutations were detected in mice during this study. Some have already been described previously in Europe such as the mutation A26T^29^, which confers moderate resistance to first-generation ARs and no resistance to second-generation ARs. Others are detected for the first time such as the S149N mutation, which also confers very little resistance to all ARs. These mutations were found with low allelic frequencies. Nevertheless, their presence on sites where the Y139C mutation is very frequently present is responsible for the emergence of a double mutant A26T/Y139C, which is certainly due to genetic recombination between mutated alleles. The emergence of double mutants has been described in France^29^, but this specific double mutant is described for the first time. The emergence of double mutation confers an evident benefit for mice carrier of these double mutations compared to mice carrier of the corresponding single mutations. While the single A26T or Y139C mutations lead to moderate or severe resistance to first generation ARs, the double mutations lead to resistance to all ARs currently available with resistance factors reaching levels higher than 10 towards difenacoum, brodifacoum and difethialone. Such levels of resistance to such molecules will certainly complicate mice management if double-mutants frequency becomes greater in a species largely associated with leptospirosis transmission (*L. interrogans*, *L. kirschneri*, *L. borgpetersenii*) to humans and domestic animals^37,38^.

Interestingly, another group of A48T/R61L mutations was also detected in an individual in the homozygous state. These mutations have been described in the introgression of the Spretus mouse *Vkorc1* gene into the domestic mouse genome that has occurred in Europe^39^. This introgression led to the introduction into the genome of the domestic mouse of a group of 4 R12W/A26S/A48T/R61L coding mutations resulting in a very strong resistance to first generation AR. In Europe, mice with this group of 4 mutations are very frequently detected, but also, mice with either one, two, or three of these mutations^29,39^. Surprisingly only 2 of these 4 mutations were detected on the island of Martinique. The presence of these mutations in Martinique may be due either to point mutations of the *Vkorc1* gene or to the importation of introgressed domestic mice from Europe. This last hypothesis should be accompanied by the presence in Martinique of domestic mice with the group of 4 characteristic mutations of the Spretus mouse genome associated with a strong resistance to first generation AR. Any additional conclusions should be based on the search in Martinique for additional traces of the *spretus* genome

For brown rats, no mutation of *Vkorc1* was found in our sample. As the brown rat population appeared to be limited in Martinique, our sampling included only 16 brown rats for which *Vkorc1* was sequenced and might not be sufficiently representative. Nevertheless, if mutations of *Vkorc1* are present in the brown rat, their frequency might be rather limited contrary to what has been described in Europe^20,30^. The use of second-generation molecules that are certainly consumed in sufficient quantities by brown rats, in urban as in agricultural, has certainly allowed to avoid the selection of resistant alleles.

For black rats, only 3 coding mutations were detected in the *Vkorc1* gene in our sample, the H68N, A115T and S149N mutations. They had never been described in previous studies^24,33–35^. These new mutations seem to induce moderate (resistance factor less than 4) or no resistance to neither the first-generation nor the second-generation molecules. Moreover, these mutations have been found only in the heterozygous state with very low allelic frequencies and never together on a single site. The black rats of Martinique therefore do not seem to present target resistance such as that described in the brown rat or the house mouse. Nevertheless, the black rat being frugivorous, they consume certainly baits in a very limited way. The low degree of resistance induced by the H68N, A115T and Y129N mutations could thus induce an inefficiency or limited efficiency of baits if present in the homozygous state. It is therefore essential to have correct treatment practices to avoid selecting these mutations and thus increasing their allelic frequency.

In conclusion, this study enables the first-time description of rodent resistance to ARs in French West Indies where leptospirosis is an endemic disease so prevalent. This resistance is clearly present with high frequency in house mouse, species which are more and more associated with leptospirosis transmission (*L. interrogans*, *L. kirshneri*, L. borgpeterseni) to humans and domestic animals^37,38^. Mice management in Martinique should be carried out carefully to prevent diffusion of resistance to first and second generation ARs and to avoid selection/emergence of new *Vkorc1* mutants or double-mutants. In black rats, even if two new *Vkorc1* mutations have been reported in this study, target resistance to ARs does not currently seem an important issue for the management of this species in Martinique. Nevertheless, other resistance mechanisms to ARs due to metabolism modification^40–42^ could exist and this study did not explore the presence and frequency of such mechanisms in rodents of Martinique.

To efficiently control rodents in Martinique in the future, it could be of interest to establish partnerships with AR users in order to build efficient strategies adapted for each population and compatible with biodiversity issues. The use of AR should be reasoned and included in an Integrated Pest Management approach combining mechanical, biological and chemical controls. First-generation ARs should be favored for the management of brown or even black rats to minimize the risk of secondary ecotoxicity because of the absence of *Vkorc1*-based resistance. On the other hand, the management of domestic mice must be based on a precautionary use of the second-generation ARs because of the high prevalence of the Y139C mutation.

## Methods

### Ethics statement

This study is part of the integrated program of surveillance, prevention and control of leptospirosis developed by the Regional Health Agencies of Guadeloupe, Martinique and Guyana and the Interregional Epidemiology Unit Antilles-Guyana. Trappings were performed by FREDON Martinique. This study was not an “experimental procedure” as defined by the French legislation (Rural Code, Article R214–89). Therefore, this study was not subject to an ethical committee approval in France. This study complied with the European Directive 2010/63/EU governing the care and use of animals in research. Wild rodents trapped in this study did not belong to endangered or protected species.

### Study area

**Figure 7:**
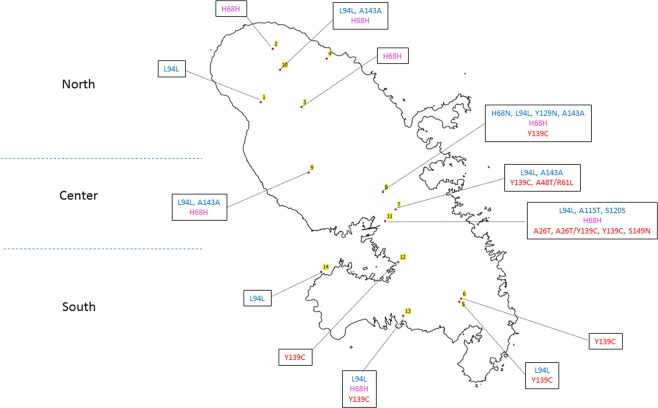
Sites of trapping in Martinique Island.

This study took place in Martinique, a French island located in the Caribbean. Thirty percent of this island is cultivated. The north is composed of hills and a volcano and different types of crops (bananas, sugar cane, cassava, pineapple…) are present. The center of the island is more urbanized. The south is tourist and composed of hills, savannas and different types of crops (Fig. 7). Field investigations conducted by FREDON Martinique have identified areas where ARs have been intensively used (ARs sites) to control rodents and in which decreases or losses of efficiency have been reported. Eight agricultural areas (sugar cane, sweet potato, cassava, banana, christophine), 2 intensive breeding sites and 2 industrial and commercial zones were selected based on these investigations for this study. Furthermore, 2 control areas without any AR use (control sites) have been also selected (Table 1 and Fig. 7). All of these sites were homogeneously distributed on Martinique Island (Central, South and North of Martinique).

### Trapping methods

Trapping in Martinique began on April 13, 2015 and ended on November 27, 2015 (Table 1). For each site, the trapping time was systematically 5 consecutive days. Depending on the size of the sites, 22 to 65 trapping stations have been set up (Table 1). These stations were homogeneously and consistently distributed on each site. The trapping stations were 10 meters apart and located in places likely to be frequented by rodents (border plots, ecotones, rivers, around livestock buildings, forest trails…). Each station included a Manufrance rat trap and an INRA trap for mice. Manufrance rat trap were baited with peanut butter, oat flakes and sardine oil, checked daily and re-baited, if necessary.

The captured rodents were killed at the trapping site by cervical dislocation, morphologically identified (*i.e*., *Rattus rattus*, *Rattus norvegicus* or *Mus musculus*), housed in an individual referenced bag and placed in a cooler with ice buns for the transport. Rodents were then kept at −20 °C until the day before their autopsy. For each rodent, a piece of tail for sequencing (1 cm maximum) and the liver to quantify ARs residues were taken and stored respectively in 70% ethanol at 4 °C and at −20 °C until sent to the laboratory.

### Rodent species identification

Species of trapped rodents were identified by morphologic aspects during autopsy. Species of trapped animals were molecularly confirmed by sequencing a portion of the mtDNA *cytochrome b* gene as described by^43^ by using specific cytb-S (5′-TCTCCATTTCTGGTTTACAAGAC-3′) and cytb-AS (5′-AACAATGACATGAAAAATCATCG TT-3′) primers. The amplified product was sequenced on both strands; the resulting sequence was submitted to blast analysis.

### Vkorc1 genotyping

Genomic DNA extracted from tail was amplified using specific primers of *vkorc1* gene as described by^29,44^ to sequence *Vkorc1* gene and detect mutations comparatively to the published *Vkorc1* sequences for *Mus Musculus domesticus* (Genbank No. GQ905710.1), *Rattus norvegicus* (Genbank No. CM000231.2) and *Rattus rattus* (Genbank No. AB702679.1).

### Heterologous expression of new VKORC1 mutants

Construction of Mm VKorc1 or RrVKORC1 mutants was carried out using pPICZ-RrVKORC1^44^ or pPICZ-MmVKORC1^29^ as template with the Quickchange site directed mutagenesis kit (Stratagene) according to the manufacturer’s recommendations. Recombinant VKORC1 proteins were produced by *Pichia pastoris* as described previously^19,29,33,38,44,45^. Yeast microsomes were obtained by differential centrifugation, as described previously^19,29,33,38,44,45^ and frozen at −80 °C until use. Protein concentrations in micrososomal fractions were determined by the method of Bradford^46^.

### Assays of VKORC1 mutant activities and kinetics

Microsomal VKOR activity was assayed as described previously^19,29,33,38,44,45^. Vitamin K production was determined by liquid chromatography-mass spectrometry.

### Determination of AR concentrations in liver

AR concentrations in liver samples were determined by the method described by^47^. The ARs detected and quantified with this method were warfarin, coumatetralyl, chlorophacinone, bromadiolone, difenacoum, brodifacoum, flocoumafen and difethialone. Limit of quantification was between 1 and 2 ng/g wet weight.

### Data analysis

A night trap effort (*i.e*., number of traps used × number of nights of trapping) was calculated for each site. Trap success (number of trapped rodents/trap effort × 100) was calculated to evaluate relative rodent abundance as described by^48^.

*K*_*i*_ values were determined in triplicate performed on two different batches of yeasts expressing the recombinant VKORC1 mutant after addition of increasing concentrations of AR (from 0.05 to 20*K*_*i*_) in the presence of different concentrations of vit K > 0 (from 0.003 to 0.2 mmol/L), as described previously^19,29,33,38,44,45^. Results were fitted by nonlinear regression to the noncompetitive inhibition model *v* = (*V*_*max*_/(1 + (*I*/*K*_*i*_))) * (*S*/(*K*_*m*_ + *S*)) using the R-fit program.

Statistical analyses were performed using GraphPad Prism 6 software using a Mann-Whitney or a Kruskal-Wallis test.

A Generalized Linear Model as a smoothing model was used to analyze the geographical pattern of ARs exposition and mutant rats/mice distribution. Binomial distribution for the response variable (proportion of rodent with ARs or proportion of mutated rodents) and logit link were used. The coordinates X and Y was used as explanatory variables. When possible, we took into account that 2 plots (sites 9 and 14) was in natural area where AR treatment was unexpected (used as control site) by adding a variable with 2 factors (variable named “Type”, levels; “natural area”, “other”). Because of the limited number of data, it was impossible to accurate the spatial trends with unlinear smoothing methods like Generalized Linear Model, however, we tested if interactions between X coordinates and Y coordinates was relevant. Variable selection was made by AIC procedure.
